# A Pathology Experience of Posttransplant Lymphoproliferative Disorder From One Tertiary Hospital: Pathology Concepts and Diagnostic Approach

**DOI:** 10.7759/cureus.54407

**Published:** 2024-02-18

**Authors:** Haneen Al-Maghrabi, Bayan Hafiz, Abdelrazak Meliti

**Affiliations:** 1 Department of Pathology and Laboratory Medicine, King Faisal Specialist Hospital and Research Centre, Jeddah, SAU; 2 Department of Pathology and Laboratory Medicine, Maternity and Children Hospital, Makkah, SAU

**Keywords:** ebv, burkitt lymphoma, diffuse large b-cell lymphoma, post-transplant lymphoproliferative disorders, lymphoma

## Abstract

Background: Solid organ transplantation and bone marrow/hematologic stem cell transplantation recipients face a heightened risk of developing malignancies or cancer as a result of immunosuppression. Posttransplant lymphoproliferative disorders (PTLD) are a range of disorders from benign lymphoid growth to lymphoma found post-transplant. Risk factors for PTLD include high immunosuppressive use and oncogenic effects of Epstein-Barr virus (EBV). There is a lack of comprehensive clinical and pathological documentation of PTLD cases among Saudi patients, and the available data are limited to a few case reports. As a result, a deeper understanding of this disease requires more clinicopathological information.

Material and Method: In this review, we share our insights on cases diagnosed with PTLD at King Faisal Specialist Hospital and Research Center, a prominent tertiary center in the western region of Saudi Arabia, from 2005-2023.

Result: We have diagnosed a total of 14 cases of PTLD in our department, with an age range spanning from 3 to 62 years. These diagnoses were made based on biopsies or tumor resection procedures. The survival rate of patients is believed to be influenced by multiple factors, including histology, tumorigenesis, disease biology, and clinical stage. Additionally, Kaplan-Meier curve analysis indicates that female patients tend to have a higher estimated survival rate compared to males.

Conclusion: PTLD diagnosis and therapy have greatly improved in the past 20 years. PTLD is treated with reduced immunosuppression, rituximab, chemotherapy, adoptive therapy, surgery, antiviral therapy, and radiotherapy. In this study, we present our experience from a large tertiary center in the western region of Saudi Arabia. Moreover, we will go through etiology, clinical features, and pathologic morphology along with the corresponding genetics, prevention, and valid treatment options.

## Introduction

Lymphoproliferative disorders (LPDs) related to induced immunodeficiency represent a range of lymphoid and/or plasmacytic growths, which includes a significant category arising after the transplantation of solid organs, stem cells, or bone marrow (BM), known as posttransplant lymphoproliferative disorders (PTLDs). LPDs associated with iatrogenic immunodeficiency occur less frequently in other scenarios, such as in patients receiving methotrexate treatment for rheumatoid arthritis or in Crohn’s disease patients who are being treated with tumor necrosis factor-α antagonists alongside antimetabolites. Not all LPDs are linked to Epstein-Barr virus (EBV), although many are. Additional clinical categorization of tumors is necessary due to their significant diversity in terms of cytology makeup, tissue destructiveness, immunophenotype expression, and cytogenetic and molecular discoveries, as well as clinical behavior and treatment modalities. Distinguishing between atypical hyperplastic lymphoid proliferation and lymphoma-like lesions in pathology is crucial. Taking this step may lead to achieving significant resolution by reducing or halting immunosuppression whenever feasible [1,2]. Correspond to the past nomenclature of the World Health Organization (WHO) classification categories, PTLD is categorized as follows: (1) nondestructive PTLD lesions, which comprise plasmacytic hyperplasia, infectious mononucleosis (IM), and florid follicular hyperplasia; (2) polymorphic PTLD, which includes polyclonal and monoclonal subtypes; (3) monomorphic PTLDs, which are classified according to lymphoma histology, resembling B-cell neoplasms such as diffuse large B-cell lymphoma (DLBCL), Burkitt lymphoma (BL), plasmablastic lymphoma (PBL), plasma cell myeloma, or other T-cell neoplasms and NK lymphomas; or (4) classic Hodgkin lymphoma (CHL) PTLD [3].

## Materials and methods

In our study, which was approved by the Research Committee of the Ethics and Research Unit, King Faisal Specialist Hospital and Research Center (IRB 2023-155), we retrospectively analyzed hematoxylin and eosin-stained slides of tumors obtained from archives at King Faisal Specialist Hospital and Research Center. The slides were collected from 14 patients diagnosed with PTLD between 2005 and 2023. We collected clinical and pathological data from patient records and diagnosed the tumors based on the fifth edition of the WHO classification of hematopoietic and lymphoid tissue tumors (Table 1). All these patients were diagnosed for the first time as PTLD after transplantation of various organs and tissues. The research data that have been gathered consist of various parameters such as patient’s age at the time of presentation, gender, site involved, PTLD pathologic subtype, EBV status, BM involvement, therapy offered, and clinical outcome. Immunohistochemistry slides for each submitted case underwent evaluation, and additional markers were applied to the chosen cases. The essential panel of immunohistochemistry ancillary studies consisted of a concise set of markers, including cluster of differentiation (CD) 45, CD20, CD5, CD3, BCL-2, BCL-6, CD10, MUM1, CD15, CD30, and the Ki-67 proliferative index. All interpretations were accurately made while ensuring the integrity of the internal controls. Monomorphic T-cell lymphomas were characterized by the inclusion of additional T-cell markers such as CD4, CD8, CD7, and T-cell receptor gene rearrangement using polymerase chain reaction. EBV in-situ hybridization (ISH) studies were done on all cases at the time of diagnosis. Selected cases included the addition of extra tests, which were fluorescence ISH for MYC and *BCL2 *and/or *BCL6 *gene rearrangements to exclude high-grade B-cell lymphomas with MYC/BCL2 and MYC, BCL2, and BCL6 rearrangements; which all were negative. According to the WHO, the cases were classified as one of four groups: nondestructive, polymorphic, monomorphic, or CHL PTLDs.

**Table 1:** : WHO categorization of posttransplant lymphoproliferative disorders (PTLD). WHO: World Health Organization The image is adapted with permission from Swerdlow et al. [3].

Category	Subtypes
Nondestructive PTLD	Plasmacytic hyperplasia, infectious mononucleosis, florid follicular hyperplasia
Polymorphic PTLD	Polyclonal (rare), monoclonal
Monomorphic PTLD (Classify according to the lymphoma they resemble)	Diffuse large B-cell lymphoma not otherwise specified, Burkitt's lymphoma, plasma cell myeloma, plasmacytoma, peripheral T-cell lymphoma not otherwise specified, hepatosplenic T-cell lymphoma
Classical Hodgkin's Lymphoma PTLD	Nodular sclerosis, mixed cellularity, lymphocyte rich, lymphocyte depleted

## Results

Table 2 summarizes the clinicopathological characteristics of the 14 cases of PTLD that were identified at our institution. All these cases were diagnosed based on biopsies or tumor resections submitted to our pathology department. The age range was between 3 and 62 years, with a median and mean age of 23.5 years and 27.5 years, respectively. There were nine males (64%) and five females (35%). Nine patients had previously undergone renal transplants (64%), four had received BM transplants (30%), and one patient had undergone a liver transplant (7%). The duration between the transplant history and the detection of the lesion ranged from 2 months to 7 years. A total of seven patients showed lymph node lesions (53%), two individuals had kidney lesions in the transplanted kidney (15%), one presented a liver lesion (7%), another had multiple gastrointestinal involvement (7%), one showed disseminated peritoneal mass (7%), one had a lesion at the base of the tongue (7%), and one displayed multiple bone lytic lesions with adjacent soft tissue involvement (7%). PTLD was diagnosed in seven patients, with 53.8% of them having the DLBCL subtype, 23% with CHL, 7.6% with BL, 7.6% with PBL, 7.6% with monomorphic T-cell lymphoma, and 7.6% with atypical paracortical lymphoid hyperplasia. Most of these patients were EBV-ISH positive (76.9%). Out of the total number of patients, five of them (35.7%) were detected to have BM involvement. The single case diagnosed with atypical paracortical lymphoid hyperplasia was controlled with sirolimus, with no disease recurrence for 9 years. Doxorubicin, bleomycin, vinblastine, and dacarbazine (ABVD) was administered to three patients for CHL treatment, two of whom received it in combination with rituximab therapy. After a follow-up of 15 and 5 years, respectively, neither showed any signs of disease recurrence. Three cases of PTLD with DLBCL were successfully treated with rituximab, cyclophosphamide, doxorubicin hydrochloride, vincristine sulfate, prednisone (R-CHOP), showing no disease recurrence during a 3-9-year follow-up period. The cases diagnosed with PTLD, a monomorphic T-cell, showed improvement with decreased dose of immunosuppressive medication. CellCept (mycophenolate mofetil) was discontinued, and the cyclosporin dose was decreased from 75 mg twice a day to 50 mg twice a day, and then decreased gradually to 25 mg in the morning and 50 mg at night. The patient was kept on prednisolone 5 mg. Unfortunately, three patients; two with PTLD-DLBCL, and one with BL, tragically passed away within a year due to their worsening health conditions. This included multiorgan failure caused by chemotherapy, cardiopulmonary failure, and worsening renal function, as documented in Table 2. The patient diagnosed with PBL showed various bone lytic lesions along with the involvement of nearby soft tissues, a frontal mass lesion, a pelvic mass, and multiple lymphadenopathies. She had cardiac arrest, anoxic encephalopathy, and was on mechanical ventilation; her Glasgow Coma Scale (GCS) was around 6 when she was off sedation. The patient received prephase (cyclophosphamide, Velcade, and steroids), continued to receive supportive care management, and, due to her poor medical condition, was not a candidate for chemotherapy. Unfortunately, the patient passed away due to cardiac arrest and multiple organ failure.

**Table 2:** Summary of posttransplant lymphoproliferative disorders (PTLD) cases. EBV-ISH: Epstein–Barr virus-in-situ hybridization; BM: bone marrow; LN: lymph node; HLH: hemophagocytic lymphohistiocytosis; HCV; hepatitis C virus; CHL: classical Hodgkin lymphoma; DLBCL: diffuse large B-cell lymphoma; BL: Burkitt lymphoma; PBL: plasmablastic lymphoma; ABVD: doxorubicin, bleomycin, vinblastine, and dacarbazine; EPIC: early postoperative intraperitoneal chemotherapy; R-CHOP: cyclophosphamide, doxorubicin, prednisone, rituximab, and vincristine; REPOCH: rituximab, etoposide phosphate, prednisone, vincristine sulfate (Oncovin), cyclophosphamide, and doxorubicin hydrochloride (hydroxydaunorubicin); HDMTX: high-dose methotrexate; GCS: Glasgow Coma Scale; ARPKD: autosomal recessive polycystic kidney disease

	Age/Sex	Previous History	Site of Involvement	Diagnosis	EBV-ISH	BM Status	Follow-Up (Years)	Treatment	Outcome
1	7 years/M	HLH/BM transplant 5 years ago	Splenic hilar LN, then left cervical LN	PTLD, CHL	Positive	Involved	NA	6-cycles of ABVD	NA
2	57 years/M	HCV/liver transplant	Liver mass	PTLD, DLBCL	NA	NA	NA	NA	NA
3	12 years/M	BM transplant 2 months ago	Left inguinal LN	PTLD, CHL	Positive	Not involved	15	ABVD + EPIC + Rituximab	No recurrence
4	62 years/F	Renal transplant 2 years ago	Left cervical LN	PTLD, monomorphic T-cell	Negative	NA	NA	Improvement with decrease in immunosuppressant medications	No show
5	26 years/F	HCV/renal transplant 1 year ago	Left kidney	PTLD, DLBCL	Positive	Involved	9	R-CHOP	No recurrence
6	3 years/M	HLH/BM transplant 2 years ago	Right cervical LN	Atypical paracortical hyperplasia	Negative	Not involved	9	Controlled on sirolimus	No recurrence
7	10 years/M	Renal transplant 2 years ago	Left cervical LN	PTLD, DLBCL	Positive	Not involved	8	R-CHOP	No recurrence
8	61 years/M	Renal transplant 7 years ago	Transplanted kidney mass	PTLD, DLBCL	Positive	NA	0	Rituximab	Death due to DIC/Multiorgan failure secondary to chemotherapy
9	8 years/M	Steam cell transplant and combined immunodeficiency	Lymph node in the back	PTLD, CHL	Positive	Involved focally	5	5 cycles of ABVD-PC + Rituximab	No recurrence
10	57 years/M	Renal transplant	Duodenum - Jejunum, and anal mass	PTLD, DLBCL	Positive	Not involved	0	Rituximab weekly for 4 doses only	Death due to Cardiopulmonary failure
11	23 years/F	Renal transplant 6 years ago	Peritoneal mass	PTLD, BL	Positive	Involved	0	6 cycles DA-REPOCH with 3 cycles HDMTX (DAY 15 f cycle 1, 3, and 6)	Death due to worsening renal profile
12	5 years/M	ESRD due to ARPKD, Status post kidney transplant 1 year ago	Base of tongue	PTLD, DLBCL	Positive	Not involved	3	R-CHOP	No recurrence
13	27 years/F	Renal transplant 7 years ago	Left cervical LN	PTLD, CHL	Positive	NA	NA	NA	No show
14	31 years/F	Renal transplant 7 years ago, on immunosuppressives	Multiple lytic bone lesions, frontal mass lesions, and multiple lymphadenopathy	PTLD, PBL	Positive	Involved	3 months	Received prephase (cyclophosphamide, Velcade, steroid), she continues supportive care	Anoxic encephalopathy, on mechanical ventilation, on propofol GCS around 6 when she is off sedation. Not candidate for chemotherapy due to critical condition

**Figure 1:**
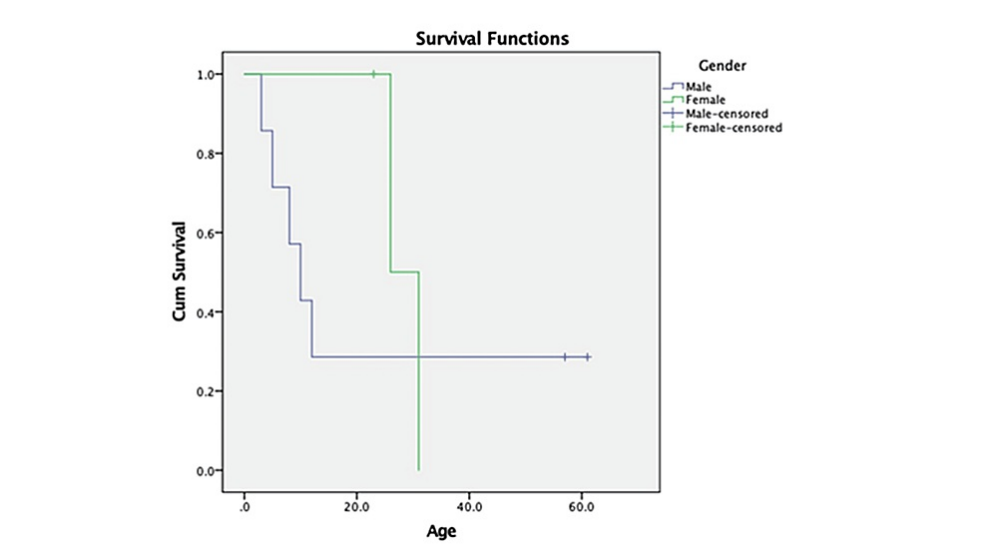
Kaplan-Meier curves showed that the 5-year overall survival for patients diagnosed as posttransplant lymphoproliferative disorders (PTLD) with a median survival of 34% (n=10).

Our research has revealed that patients aged 3-26 years old have demonstrated no recurrence and have achieved a high overall survival rate. Despite undergoing intensive therapy, the young patient diagnosed with PTLD, BL, and disseminated disease with aggressive histology did not survive. We strongly believe that the survival rate of patients is influenced by various factors, such as the histology and tumorigenesis biology of the disease, along with the clinical stage. Although a better-estimated survival rate in women compared to men was suggested, the differences were not statistically significant (p=0.54) (Figure 1).

## Discussion

Epidemiology and etiology

The frequency of PTLDs varies greatly depending on the organ that has been transplanted, but it is estimated that approximately 2% of all transplant recipients will experience the development of PTLDs. The rejection rates for organ transplants vary widely: kidneys have a rejection rate of 0.5-2.5%; BM 1-2%; liver 1-10%; heart and lungs 2-10%; and intestinal and multivisceral organs 5-20% [4-7]. The risk of developing PTLD is higher in young people, particularly during the initial phases of the disease. After the age of 50, the risk increases once again [6,8]. On the whole, men are more likely to experience PTLD, especially in cases of late-onset PTLD, whereas women are more likely to develop PTLD after receiving a small bowel transplant [6,9]. The incidence of PTLD can be impacted by numerous factors. The absence of EBV antibodies at the time of organ transplantation is a significant risk factor that contributes to the higher occurrence of PTLD in pediatric populations compared to adults [8]. The incidence of PTLD increases by 10- to 75-fold when transplantation occurs from a donor who is EBV-seropositive to a recipient who is EBV-seronegative (known as EBV mismatch) [10]. Increased occurrence of PTLD is also linked to a lack of previous viral exposure to cytomegalovirus (CMV) antigen. Moreover, some studies indicate that patients who receive transplantation for liver cirrhosis induced by the hepatitis C virus are more likely to develop PTLD, which suggests that hepatitis C may enhance the cancer-causing potential of EBV oncogenes [11,12]. Host factors, including certain genetic variations and human leukocyte antigens, can influence the risk of certain PTLD cases [13-15]. Even after accounting for the serum levels of EBV and CMV, the maintenance of a preparatory immunosuppressive regimen used for a transplant patient as well as for the treatment of graft-versus-host disease represents a significant risk factor for PTLD development. The combined intensity of immunosuppressive therapy and the specific agents used are linked to the risk of early onset PTLD, whereas the overall duration of immunosuppression is associated with the risk of later onset PTLD [10,16]. Anti-T-cell antibodies such as OKT3 and ATG increase the risk of PTLD and are used to remove T-cells from BM or stem cell products before transplantation [10,16-19]. Lower chances of PTLD might be linked with certain newly developed immunosuppressive techniques [9,17]. Nevertheless, the latest immunosuppressive therapy was designed to counter the adverse effects of prolonged use of calcineurin inhibitors and to avoid nephrotoxicity. Regrettably, it may also increase the risk of developing PTLD [20]. EBV infection can occur in various ways: through primary infection from the donor, superinfection by a second EBV strain in a seropositive recipient, or reactivation of latent EBV in an adult recipient. EBV-associated PTLD latency patterns are unpredictable, and different groups of latency-expressing proteins are seen in target cells [21,22]. Most cases exhibit latency pattern III, very similar to that observed in EBV+ lymphoblastoid cells. A moderate number of cases exhibit latency pattern II, whereas a smaller number exhibit pattern I [23,24]. PTLD of the classical Hodgkin’s lymphoma type can exhibit a pattern corresponding to type II [25]. PTLDs are believed to originate as polyclonal proliferations that are associated with EBV or so-called other triggers, although very few patients show a consecutive progression of the ailment. Over time, these proliferations can develop into oligoclonal and eventually monoclonal B-cell proliferation or, in rare situations, T-cell clonal proliferations [26,27]. The development and progression of B-cell clonal proliferations in PTLDs may also be influenced by antigenic selection. Enhancing our understanding of the cellular pathways linked to PTLD development is not only a matter of academic interest, but also holds promise for innovative treatment strategies, such as the use of JAK/STAT inhibitors [28,29].

Although most of the PTLD cases are EBV-positive, in approximately 20-40% of PTLDs, EBV cannot be detected [30]. The exact reason behind EBV-negative PTLD is unclear; however, certain instances may be indicative of EBV-induced growth that no longer contains the virus after undergoing tumor genesis (as per the formerly known hit-and-run theory) [31]. Other possible causes include technical challenges in identifying viral bodies, growth triggered by different infectious or viral sources other than EBV, or persistent body exposure to antigens, conceivably from the transplant procedure itself. Some researchers have reported human herpesvirus 8 (HHV-8)-positive PTLD as polymorphic lesions, such as cases seen in CD, and primary effusion lymphoma [32,33]. Research on gene expression profiling has indicated a distinct variance in the pathogenesis of PTLDs based on their EBV status, with EBV-positive PTLDs exhibiting a different gene profile [34].

Clinical features

Previously, most PTLDs were diagnosed within the first year after transplantation. However, new research suggests that PTLD development could take several years or even longer. Research has shown a considerable variation in these results, with up to 25% manifesting more than 10 years after the transplantation [4,35]. PTLD that occurs at an early age has been linked to patients who are younger, show symptoms resembling IM, test positive for EBV, and can evolve hepatosplenic T-cell lymphoma PTLD after BM and heart-lung transplantation [18,35]. Although the literature contains inconclusive data, there are reports indicating that patients who seek treatment after a longer period are at a higher risk of localized extranodal disease and a poorer prognosis [36,37]. It has been observed that polymorphic PTLD signs and symptoms tend to appear earlier than monomorphic ones. This could be because the latter are more habitually EBV-negative [38]. PTLD may exhibit tumor mass effects, resulting in a widespread illness that resembles IM. Their symptoms may be nonspecific and undefined, such as fever, or they may be entirely asymptomatic. The gastrointestinal tract (often multifocal involvement), lymph nodes, lungs, and liver are the most frequently affected. PTLDs affecting the central nervous system typically display a monomorphic nature, have poor prognosis, and tend to manifest quite late after transplantation, despite frequently exhibiting EBV positivity [39]. Enlargement of tonsils or adenoids is frequently noticed in initial PTLD lesions, whereas lymph node involvement tends to develop more in adults. Younger patients often show symptoms similar to IM [40]. The potential for monomorphic plasmacytoma lesions to be either localized or disseminated disease is correlated with increased levels of body para protein, lactate dehydrogenase, and beta2-microglobulin [41]. PTLDs can present as an aggressive and widespread disease, which includes plasma cell myeloma/neoplasms. These cases are often seen in older individuals and those who have undergone a BM transplant. This may pose a challenge to pathologists and clinicians in differentiating it from transplant rejection.

An increase in EBV viral load can often be observed, sometimes even before the appearance of visible symptoms; thus, surveillance is suggested as a method for monitoring patients at high risk [42]. It is important to acknowledge that EBV+ PTLD may still occur in circumstances where viral loads are low. Only in certain situations, persistently high levels suggest an elevated risk of PTLD, and these levels may spontaneously resolve. Moreover, some studies indicate that the type of immunosuppressive treatment or the extent of iatrogenic immunosuppression reagents may have more of an impact on EBV load [43]. In conclusion, there is currently no agreement on standardized methodologies or a standard care protocol for measuring viral load. Moreover, it is still unclear how these findings should be incorporated with other diagnostic markers.

Pathology

PTLDs include a wide range of lesions, varying from nondestructive early proliferations to more invasive and tissue-damaging lesions. These lesions can be either polymorphic or monomorphic proliferations, as shown in Table 1. Pathologists face significant challenges when categorizing PTLDs based on morphological characteristics, especially when encountering borderline cases that rely heavily on subjective interpretation. This is mainly due to the frequent fluctuations in the lesions and differences observed among various regions of the ailment. Roughly up to 80% of PTLD cases are classified as monomorphic type. EBV+ cases are mostly associated with geographic tumor necrosis [44]. Around 30% of the BM is impacted. They tend to be more prevalent in individuals with monomorphic PTLD-DLBCL type [45]. Nondestructive PTLD can be manifested as plasmacytic hyperplasia, typically seen in lymph nodes or tonsils that reveal an increased proliferation of small polytypic lymphocytes and plasma cells, not uncommon when few transformed (larger) cells can be seen; yet the overall structural architecture integrity is maintained. These changes are not considered PTLD, and they are often inseparable from reactive lymphoid hyperplasia, particularly in the absence of EBV. IM-PTLD is typically identified in tonsils, adenoids, and lymph nodes. They exhibit a mixed proliferation of small lymphocytes, plasma cells, histocytes, eosinophils, and frequently highly noticeable transformed immunoblasts or binucleated Reed-Sternberg (RS)-like cells. It should be noted that the fundamental structure of the lymph node or tonsil remains undamaged, although IM-PTLD and polymorphic PTLD located in tonsils may be indistinguishable in florid cases. Ensuring the absence of partial nodal involvement by a monomorphic PTLD is crucial in these cases. According to the WHO classification, it is recognized that certain nondestructive PTLDs can showcase a remarkable increase in follicular hyperplasia, without the usual nonfollicular effect seen in other types of PTLDS [3,38,40]. It is vital to acknowledge that caution is necessary when there is no significant EBV positivity or genuine mass lesion because the findings of follicular hyperplasia are entirely nonspecific.

**Figure 2:**
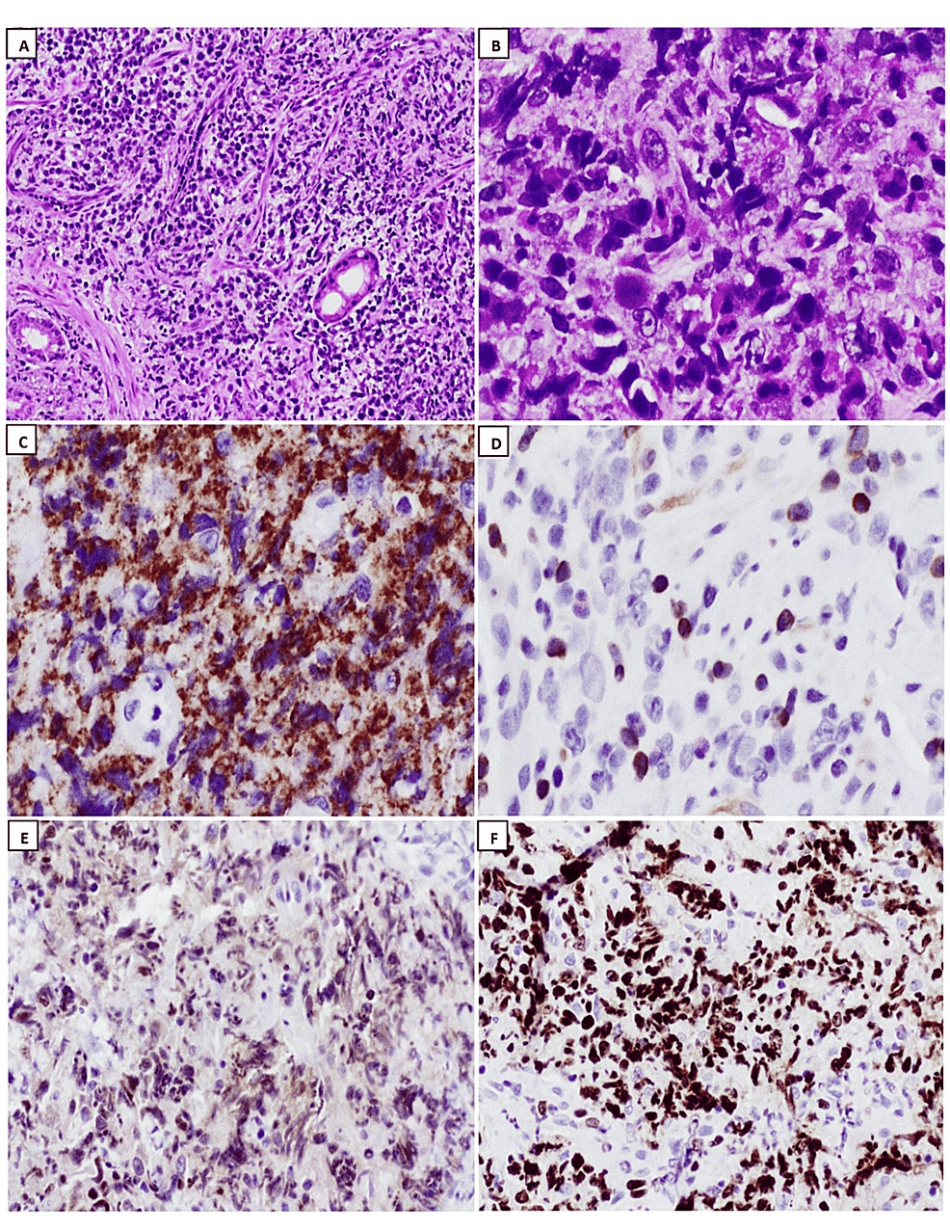
Histopathology exanimation: hematoxylin and eosin stain (H&E) and immunohistochemistry studies of diffuse large B-cell lymphoma (DLBCL). (A): low-power field examination revealed infiltrative lymphoid neoplastic cells distracting normal intestinal glands (H&E; 4x), (B): sheets of large neoplastic cells with cytoplasm, clumped chromatin and prominent nucleoli (H&E; 40x), (C): atypical cells are diffuse and strongly positive for CD20 (40x), (D): scattered atypical cells are staining for BCL2 (40x), (E): BCL6 positive in tumor cells (20x), (F): Ki67 proliferative index is expressed in around 90% of tumor cells (20x).

**Figure 3:**
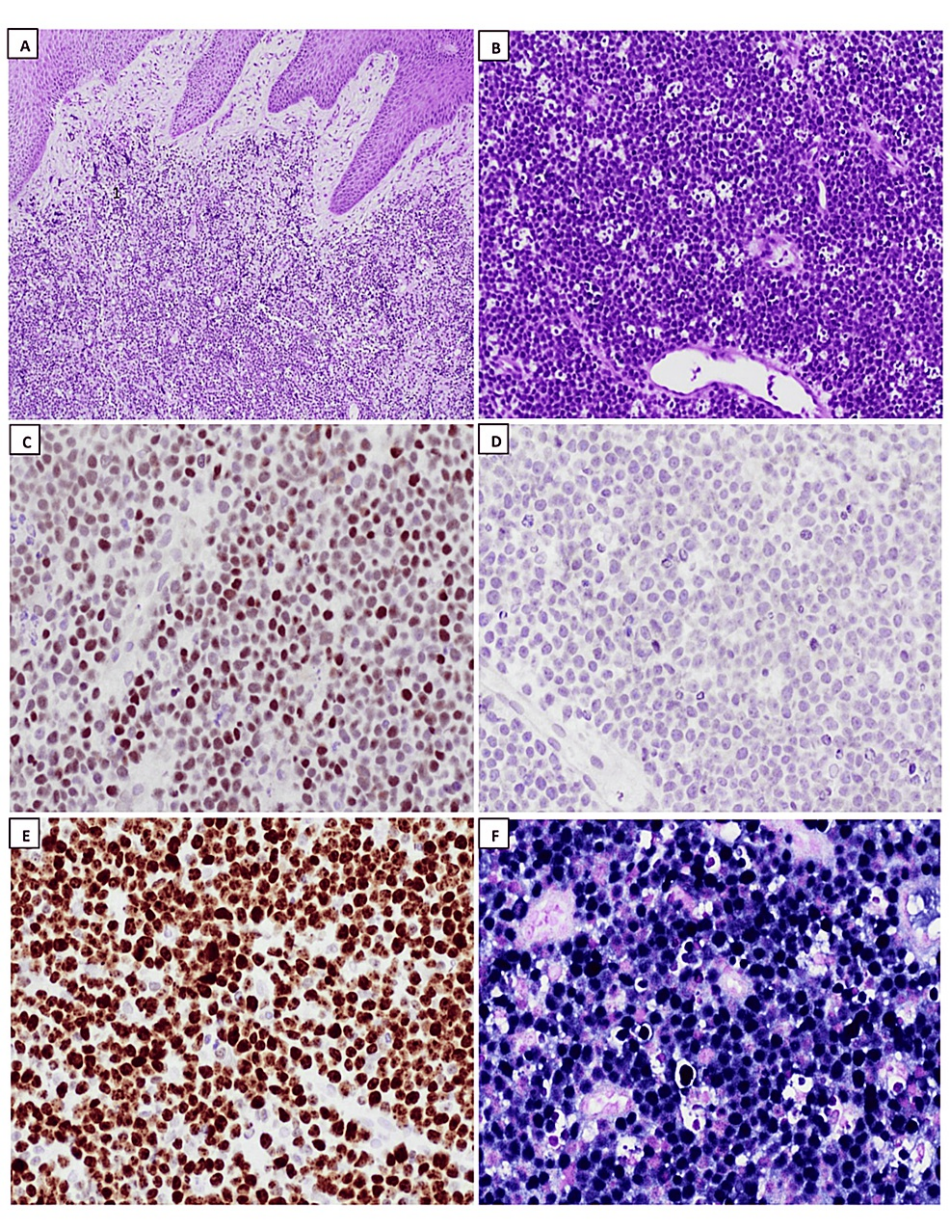
Histopathology exanimation: hematoxylin and eosin stain (H&E) and immunohistochemistry studies of Burkitt lymphoma (BL). (A): low-power field examination revealed squamous epithelial lining with underling infiltrative lymphoid neoplastic cells (H&E; 4x), (B): sheets of neoplastic infiltration of monomorphic, medium size cells with abundant basophilic cytoplasm, non-cleaved round nuclei with coarse chromatin and distinct nucleoli forming starry sky pattern (H&E; 10x), (C): neoplastic cells are positive for BCL6 (20x), (D): neoplastic cells are negative for BCL2 (20x), (E): Ki67 proliferative index is expressed in more than 99% of tumor cells (20x), (F): tumor cells expressing strongly diffuse positive EBV staining (20x).

**Figure 4:**
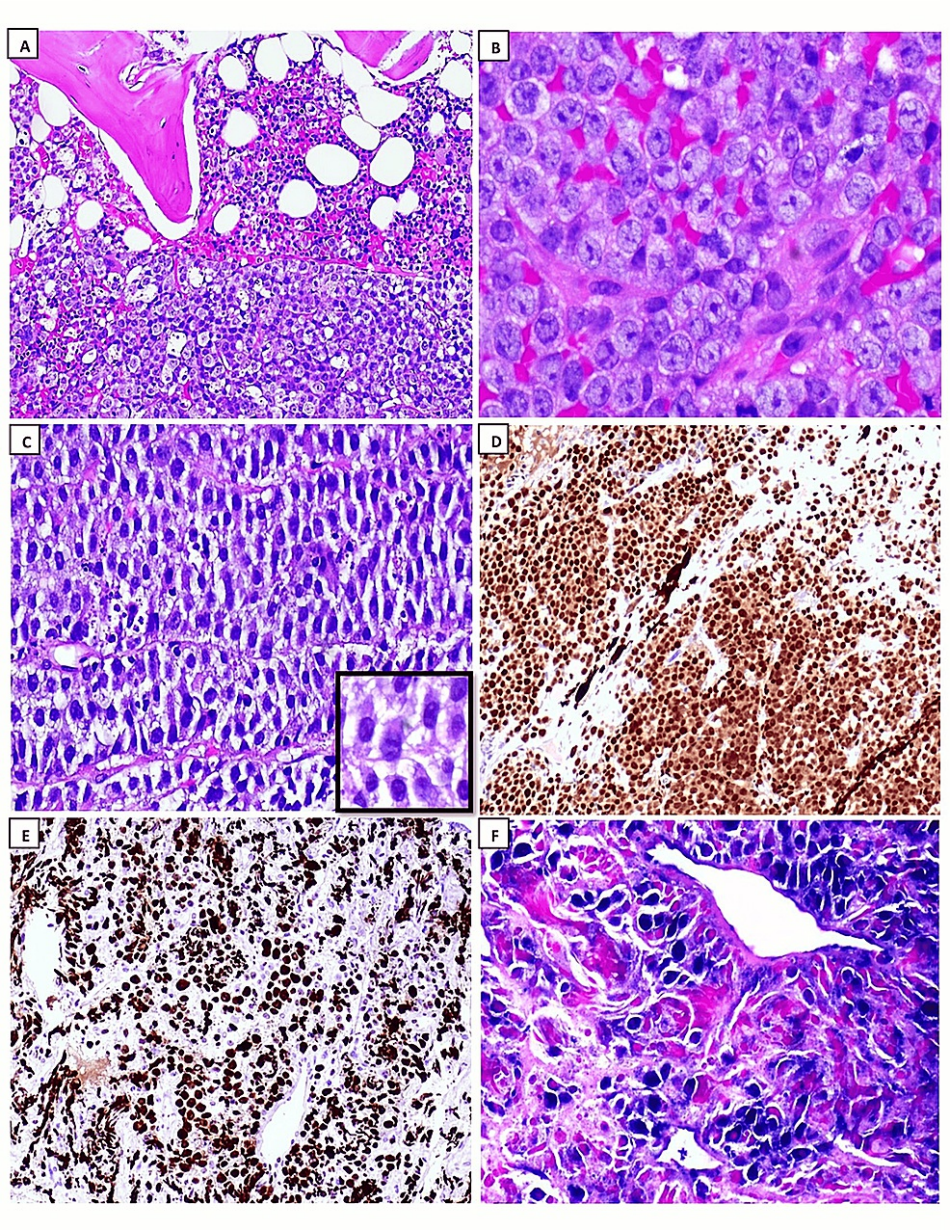
Histopathology exanimation: hematoxylin and eosin stain (H&E) and immunohistochemistry studies of plasmablastic lymphoma (PBL). (A): low-power field examination revealed bone marrow with underling infiltrative lymphoid neoplastic cells, starry sky pattern focally seen (H&E; 4x), (B): sheets of neoplastic infiltration in bone marrow composed of intermediate to large sized cells with immunoblastic looking cells and prominent nucleoli, note the atypical mitosis (in the middle of the picture) (H&E; 40x), (C): sheets of neoplastic cells in soft tissue composed of eccentric cytoplasm, plasmacytic differentiation (inset) (H&E; 20x), (D): neoplastic cells are diffusely positive for MUM1 (10x), (E): Ki67 proliferative index is expressed in more than 99% of tumor cells (20x), (F): tumor cells expressing strongly diffuse positive EBV staining (40x).

**Figure 5:**
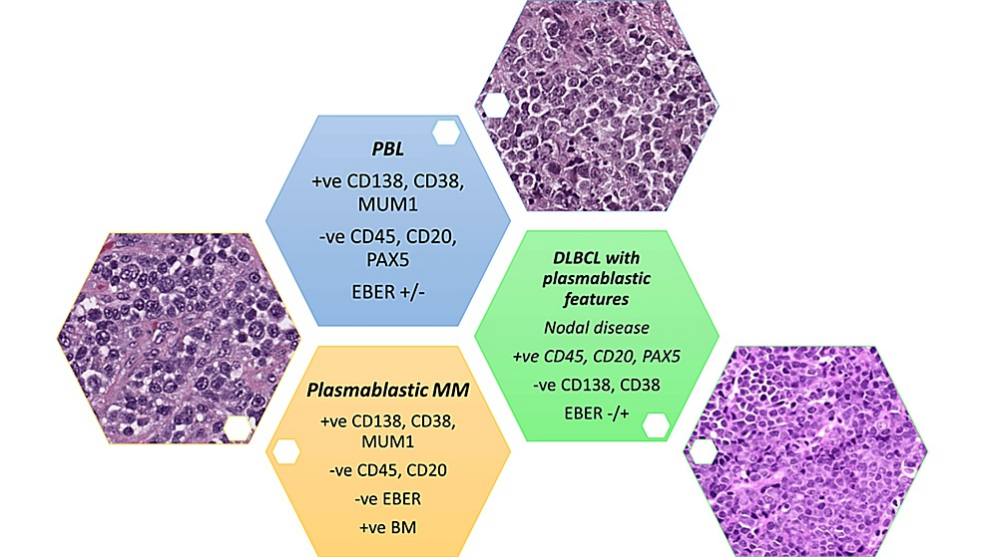
Diagnostic dilemma between plasmablastic lymphoma (PBL), plasmablastic/anaplastic plasma cell myeloma (MM), and diffuse large B-cell lymphoma (DLBCL) with plasmablastic features. +ve: positive; -ve: negative; EBER: Epstein–Barr virus-encoded small RNAs; BM: bone marrow

Polymorphic PTLD is a disorder characterized by uncontrolled destructive growth of lymphocytes, plasma cells, and immunoblasts, which vary in size and shape. The small lymphocytes were thought to resemble germinal center cells because of the angular or cleft-like shape of their nuclei. Immunoblasts can be multinucleated with highly prominent nucleoli. In the past, polymorphic PTLD was often misdiagnosed as Hodgkin-like PTLD (a denomination that is no longer acknowledged). Therefore, immunohistochemistry ancillary studies are necessary to differentiate between the two and identify cases of CHL-PTLD. In certain instances, there is evidence of extensive necrosis covering considerable geographical regions, which typically coincide with the presence of neutrophils and histiocytes and are encircled by a heightened population of altered cells or immunoblasts. Apoptosis may also manifest itself. If a case displays a dominance of transformed cells or immunoblasts, even if they appear pleomorphic, or if there are prominent transformed cells with differentiation from mature plasma cells, or if the case fulfills the criteria for a T-cell/histiocyte-rich large B-cell lymphoma or for a T-cell lymphoma, the diagnosis of polymorphic PTLD should not be applied. Furthermore, researchers applying the analysis of gene expression have not clearly differentiated between polymorphic PTLD and non-germinal center types of monomorphic PTLD, suggesting that this distinction may be difficult and reliant on subjective criteria [38,44,46]. Certain PTLDs meet the standard criteria for EBV+ mucocutaneous ulcers (MCUs) [47]. These cases present as oral or gastrointestinal tract ulcerations that are well-defined (cup-shaped) and exhibit varying degrees of polymorphism. The target large B-cells are positive for CD30 and EBV, and some of these cells may resemble RS-like cells. Another helpful clue is that T-cells are present and tend to form a band at the base of the infiltrate. Interestingly, the presence of B-cell clonality can be detected in some instances, but not in every case. However, monoclonal T-cell populations can be found in most MCUs [48]. Lesions can disappear with reduced immunosuppression, with or without rituximab and without changing therapy [47]. Monomorphic PTLDs refer to lymphoid or plasmacytic growths that meet the necessary requirements for either non-Hodgkin’s lymphoma or plasma cell malignancy in individuals with a functional immune system. These growths must be pathologically classified based on the neoplastic subtype they closely simulate. English literature reviews showed that the majority of monomorphic PTLDs consist of DLBCL subtypes (Figure 2). BL (Figure 3) is a less common type found in 5% of PTLDs. Occasionally, PBL (Figure 4) cases may be present, as seen in our collected data. Monomorphic EBV+DLBCLs are commonly found in elderly individuals. It can be considerably challenging and somewhat subjective to differentiate between polymorphic PTLD and monomorphic PTLD of the DLBCL subtype. There is a lack of definite criteria when it comes to identifying borderline lesions. Cases with mixed monotypic plasma cells and transformed B-cells or immunoblasts can be challenging when the latter cells are not the predominant population. Like polymorphic PTLD, B-cell maturation varies, but in non-immunocompromised individuals, these cases meet malignant lymphoma standards, like monomorphic PTLD [49]. Plasma cell myelomas are infrequent and must satisfy all standards for plasma cell myeloma in a healthy individual. Although some cases exhibit prominent nucleoli that are not aggressive, they are still considered as possibly having plasmablastic-type PTLD. Our study reveals one case diagnosed with PBL, which is an aggressive lymphoma that displays plasmablast or immunoblast morphological traits and exhibits differentiation toward plasma cells. The main differential diagnosis in these clinical situations is plasmablastic/anaplastic plasma cell myeloma. The patient’s history of posttransplant EBV-positive lymph nodes favors PBL over multiple myeloma. Moreover, MYC gene rearrangement is more supportive of PBL, which can be found in approximately 50% of instances [50]. ALK and HHV-8 should be negative in these instances. It is important to note that the diagnosis of these cases depends heavily on correlating the patient’s clinical history with laboratory findings. Nevertheless, it is vital to acknowledge that distinguishing between PBL and plasmablastic/anaplastic myeloma may not always be feasible (Figure 5). Cutaneous and subcutaneous B-cell PTLDs, which represent 38% of all cutaneous cases, share characteristics with a type of EBV-positive extranodal marginal zone lymphoma of mucosa-associated lymphoid tissue (MALT) lymphoma that shows plasmacytic differentiation. This lymphoma mainly affects the skin and subcutaneous tissues, appears as solitary growth after transplantation, and usually has a good prognosis [51,52]. MALT lymphomas have also been identified in the stomach and salivary glands, although infrequently, among posttransplant patients, they are typically lacking EBV [53].

Fewer than 15% of PTLDs are attributed to T-cells or rare NK-cell PTLDs, which are monomorphic [54]. T-cell PTLDs may not have larger transformed cells like most monomorphic B-cell PTLDs and can look like a healthy host. T-cell gene rearrangement and immunohistochemistry are crucial for T-cell PTLD suspicion because these cases can resemble polymorphic PTLDs. Most cases meet the requirements for peripheral T-cell lymphoma, not otherwise specified (NOS). About 15% of hepatosplenic T-cell lymphomas occur after transplant, constituting 10% of all reported T-cell PTLDs. Rare aggressive NK cell neoplasms can occur. It is important to differentiate it from posttransplant T-cell granular lymphocytic leukemia. There have been reported instances of T-lymphoblastic leukemia/lymphoma, which are uncommon, including a few that may be related to previous non-T-cell blastic neoplasms in a clonal manner [55]. CHL PTLD accounts for only 8% of cases and typically exhibits similarities to the mixed cellular CHL subtype [56]. To be classified as CHL, the case must meet the requirements of both morphological and immunophenotypic criteria. This is particularly important because PTLDs often contain atypical immunoblasts or RS-like cells, and “Hodgkin-like” diagnoses are no longer considered part of this group.

Genetics and molecular

Immunoglobulin gene rearrangement and EBV terminal analysis can demonstrate that the majority of B-cell polymorphic and monomorphic PTLDs are monoclonal in nature. In the same patient, lesions can show different B-cell clones or a single clone in one location and multiple clones in another [57]. It is possible that recurrent PTLD could indicate the existence of identical or separate clones to the previous ones [27]. In most cases of monomorphic T-cell lymphoma PTLDs, genotypic studies have confirmed the presence of T-cell clonality. However, in some instances, both clonal B-cells and T-cells have been observed either at the same time or in different lesions [58]. Caution should be exercised when dealing with B-cell PTLDs because about 50% demonstrate monoclonal T-cell populations. In cases with a CD8+ T-cell predominance and no identifiable T-cell PTLD. The discovery of these non-neoplastic restricted T-cell populations in a significant number of B-cell PTLDs indicates their frequent occurrence [59]. Cytogenetic abnormalities that are frequently reported include trisomy 9, trisomy 11, MYC rearrangement at 8q24, IGH-gene at14q32, and disruption in 1q11-21. Rearrangements of the *BCL2 *and *BCL6* genes have been reported in a few cases of PTLD, as well as in a limited number of cases where there are gains in *MYC*, *BCL2*, and *BCL6* genes [60]. Nearly half of all PTLD cases were found to have a deficiency in displaying the cyclin-dependent kinase inhibitor p16/INK4a. This deficiency was particularly associated with predominantly monomorphic or EBV cases and those with a higher proliferative index [61]. A significant percentage of T-cell PTLD cases have been found to exhibit mutations in TP53 and other oncogenes [62].

The study is limited by the small sample size because the disease is rare. Additionally, several patients were lost during the follow-up period, which impacts the overall long-term follow-up of the sample size.

## Conclusions

Receiving solid organ transplantation or BM/hematologic stem cell transplantation puts individuals at a higher risk of developing cancer due to the suppression of their immune system. PTLD encompass a range of conditions, from benign lymphoid growth to lymphoma, that occur after transplantation. Factors that increase the risk of PTLD include frequent use of immunosuppressive medications and the oncogenic effects of EBV. Unfortunately, comprehensive clinical and pathological documentation of PTLD cases among Saudi patients is lacking, with only a limited number of cases reports available. Consequently, there is a need for more clinicopathological information to gain a deeper understanding of this disease. Here we present a unique study from a tertiary cancer center along with a literature review.

## References

[1] Lewin KJ (1997). Post-transplant lymphoproliferative disorders. Pathol Oncol Res.

[2] Swerdlow SH (1997). Classification of the posttransplant lymphoproliferative disorders: From the past to the present. Semin Diag Pathol.

[3] Swerdlow SH, Campo E, Harris N L (2008). WHO classification of tumours of haematopoietic and lymphoid tissues. Vol. 2. http://apps.who.int/bookorders/anglais/detart1.jsp?codlan=1&codcol=70&codcch=4002.

[4] Evens AM, Roy R, Sterrenberg D, Moll MZ, Chadburn A, Gordon LI (2010). Post-transplantation lymphoproliferative disorders: Diagnosis, prognosis, and current approaches to therapy. Curr Oncol Rep.

[5] Neuringer IP (2013). Posttransplant lymphoproliferative disease after lung transplantation. J Immunol Res.

[6] Engels EA, Pfeiffer RM, Fraumeni JF (2011). Spectrum of cancer risk among US solid organ transplant recipients. JAMA.

[7] Ramos E, Hernández F, Andres A (2013). Post‐transplant lymphoproliferative disorders and other malignancies after pediatric intestinal transplantation: Incidence, clinical features and outcome. Pedia transplant.

[8] Quinlan SC, Pfeiffer RM, Morton LM, Engels EA (2011). Risk factors for early-onset and late-onset post-transplant lymphoproliferative disorder in kidney recipients in the United States. Am J Hematol.

[9] Nassif S, Kaufman S, Vahdat S, Yazigi N, Kallakury B, Island E, Ozdemirli M (2013). Clinicopathologic features of post‐transplant lymphoproliferative disorders arising after pediatric small bowel transplant. Pedia transplant.

[10] Cockfield S (2001). Identifying the patient at risk for post‐transplant lymphoproliferative disorder. Transpl Infect Dis.

[11] HEzode C, Duvoux C, Germanidis G (1999). Role of hepatitis C virus in lymphoproliferative disorders after liver transplantation. Hepatology.

[12] Buda A, Caforio A, Calabrese F (2000). Lymphoproliferative disorders in heart transplant recipients: Role of hepatitis C virus (HCV) and Epstein‐Barr virus (EBV) infection. Transpl Internat.

[13] Babel N, Vergopoulos A, Trappe RU (2007). Evidence for genetic susceptibility towards development of posttransplant lymphoproliferative disorder in solid organ recipients. Transplantation.

[14] Reshef R, Luskin MR, Kamoun M (2011). Association of HLA polymorphisms with post-transplant lymphoproliferative disorder in solid-organ transplant recipients. Am J Transplant.

[15] McAulay K, Haque T, Crawford D (2009). Tumour necrosis factor gene polymorphism: a predictive factor for the development of post-transplant lymphoproliferative disease. Br J cancer.

[16] Tanner JE, Alfieri C (2001). The Epstein-Barr virus and post-transplant lymphoproliferative disease: Interplay of immunosuppression, EBV, and the immune system in disease pathogenesis. Transpl Infect Dis.

[17] Caillard S, Dharnidharka V, Agodoa L, Bohen E, Abbott K (2005). Posttransplant lymphoproliferative disorders after renal transplantation in the United States in era of modern immunosuppression. Transplantation.

[18] Curtis RE, Travis LB, Rowlings PA (1999). Risk of lymphoproliferative disorders after bone marrow transplantation: A multi-institutional study. Blood.

[19] Brunstein CG, Weisdorf DJ, DeFor T, Barker JN, Tolar J, van Burik JA, Wagner JE (2006). Marked increased risk of Epstein-Barr virus-related complications with the addition of antithymocyte globulin to a nonmyeloablative conditioning prior to unrelated umbilical cord blood transplantation. Blood.

[20] Sam T, Gabardi S, Tichy EM (2013). Risk evaluation and mitigation strategies: A focus on belatacept. Prog Transplant.

[21] Oudejans JJ, Jiwa M, van den Brule AJ (1995). Detection of heterogeneous Epstein-Barr virus gene expression patterns within individual post-transplantation lymphoproliferative disorders. Am J Pathol.

[22] Rea D, Fourcade C, Leblond V (1994). Patterns of Epstein-Barr virus latent and replicative gene expression in Epstein-Barr virus B cell lymphoproliferative disorders after organ transplantation. Transplantation.

[23] Gonzalez-Farre B, Rovira J, Martinez D (2014). In vivo intratumoral Epstein-Barr virus replication is associated with XBP1 activation and early-onset post-transplant lymphoproliferative disorders with prognostic implications. Mod Pathol.

[24] Vase MØ, Maksten EF, Bendix K (2015). Occurrence and prognostic relevance of CD30 expression in post-transplant lymphoproliferative disorders. Leuk Lymphoma.

[25] Delecluse HJ, Kremmer E, Rouault JP, Cour C, Bornkamm G, Berger F (1995). The expression of Epstein-Barr virus latent proteins is related to the pathological features of post-transplant lymphoproliferative disorders. Am J Pathol.

[26] Muti G, De Gasperi A, Cantoni S (2000). Incidence and clinical characteristics of posttransplant lymphoproliferative disorders: Report from a single center. Transpl Int.

[27] Wu T-t, Swerdlow SH, Locker J (1996). Recurrent Epstein-Barr virus-associated lesions in organ transplant recipients. Hum Pathol.

[28] Alsayed Y, Leleu X, Leontovich A, Oton AB, Melhem M, George D, Ghobrial IM (2008). Proteomics analysis in post-transplant lymphoproliferative disorders. Eur J Haematol.

[29] Vaysberg M, Lambert SL, Krams SM, Martinez OM (2009). Activation of the JAK/STAT pathway in Epstein Barr virus+-associated posttransplant lymphoproliferative disease: Role of interferon-gamma. Am J Transplant.

[30] Perry AM, Aoun P, Coulter DW, Sanger WG, Grant WJ, Coccia PF (2013). Early onset, EBV(-) PTLD in pediatric liver-small bowel transplantation recipients: A spectrum of plasma cell neoplasms with favorable prognosis. Blood.

[31] Srinivas SK, Sample JT, Sixbey JW (1998). Spontaneous loss of viral episomes accompanying Epstein-Barr virus reactivation in a Burkitt's lymphoma cell line. J Infect Dis.

[32] Kapelushnik J, Ariad S, Benharroch D, Landau D, Moser A, Delsol G, Brousset P (2001). Post renal transplantation human herpesvirus 8-associated lymphoproliferative disorder and Kaposi's sarcoma. Br J Haematol.

[33] Dotti G, Fiocchi R, Motta T (1999). Primary effusion lymphoma after heart transplantation: A new entity associated with human herpesvirus-8. Leukemia.

[34] Morscio J, Dierickx D, Ferreiro JF (2013). Gene expression profiling reveals clear differences between EBV-positive and EBV-negative posttransplant lymphoproliferative disorders. Am J Transplant.

[35] Dierickx D, Tousseyn T, Sagaert X (2013). Single-center analysis of biopsy-confirmed posttransplant lymphoproliferative disorder: Incidence, clinicopathological characteristics and prognostic factors. Leuk Lymphoma.

[36] Nelson BP, Nalesnik MA, Bahler DW, Locker J, Fung J, Swerdlow S (2000). Epstein-Barr virus-negative post-transplant lymphoproliferative disorders: A distinct entity?. Am J Surg Pathol.

[37] Armitage J, Kormos RL, Stuart RS (1991). Posttransplant lymphoproliferative disease in thoracic organ transplant patients: Ten years of cyclosporine-based immunosuppression. J Heart lung transplant.

[38] Vakiani E, Basso K, Klein U (2008). Genetic and phenotypic analysis of B-cell post-transplant lymphoproliferative disorders provides insights into disease biology. Hematol Oncol.

[39] Evens AM, Choquet S, Kroll-Desrosiers AR (2013). Primary CNS posttransplant lymphoproliferative disease (PTLD): An international report of 84 cases in the modern era. Am J Transplant.

[40] Nelson BP, Wolniak KL, Evens A, Chenn A, Maddalozzo J, Proytcheva M (2012). Early posttransplant lymphoproliferative disease: Clinicopathologic features and correlation with mTOR signaling pathway activation. Am J Clin Pathol.

[41] Karuturi M, Shah N, Frank D (2013). Plasmacytic post-transplant lymphoproliferative disorder: A case series of nine patients. Transpl Int.

[42] Tsai DE, Douglas L, Andreadis C (2008). EBV PCR in the diagnosis and monitoring of posttransplant lymphoproliferative disorder: Results of a two-arm prospective trial. Am J Transplant.

[43] Ahya VN, Douglas LP, Andreadis C (2007). Association between elevated whole blood Epstein-Barr virus (EBV)-encoded RNA EBV polymerase chain reaction and reduced incidence of acute lung allograft rejection. J Heart Lung Transplant.

[44] Morscio J, Dierickx D, Tousseyn T (2013). Molecular pathogenesis of B-cell posttransplant lymphoproliferative disorder: What do we know so far?. Clin Dev Immunolo.

[45] Montanari F, O'Connor OA, Savage DG (2010). Bone marrow involvement in patients with posttransplant lymphoproliferative disorders: Incidence and prognostic factors. Hum Pathol.

[46] Parker A, Bowles K, Bradley JA (2010). Diagnosis of post-transplant lymphoproliferative disorder in solid organ transplant recipients - BCSH and BTS Guidelines. Br J Haematol.

[47] Hart M, Thakral B, Yohe S, Balfour HH Jr, Singh C, Spears M, McKenna RW (2014). EBV-positive mucocutaneous ulcer in organ transplant recipients: A localized indolent posttransplant lymphoproliferative disorder. Am J Surg Pathol.

[48] Dojcinov SD, Venkataraman G, Raffeld M, Pittaluga S, Jaffe ES (2010). EBV positive mucocutaneous ulcer—A study of 26 cases associated with various sources of immunosuppression. Am J Surg Pathol.

[49] Swerdlow S (1997). Post-transplant lymphoproliferative disorders: A working classification. Curr Diag Pathol.

[50] Montes-Moreno S, Martinez-Magunacelaya N, Zecchini-Barrese T (2017). Plasmablastic lymphoma phenotype is determined by genetic alterations in MYC and PRDM1. Mod Pathol.

[51] Wang E, Stoecker M (2010). Primary cutaneous giant cell plasmacytoma in an organ transplant recipient: A rare presentation of a posttransplant lymphoproliferative disorder. Am J Dermatopathol.

[52] Gibson SE, Swerdlow SH, Craig FE (2011). EBV-positive extranodal marginal zone lymphoma of mucosa-associated lymphoid tissue in the posttransplant setting: A distinct type of posttransplant lymphoproliferative disorder?. Am J Surg Pathol.

[53] Hsi ED, Singleton TP, Swinnen L, Dunphy CH, Alkan S (2000). Mucosa-associated lymphoid tissue-type lymphomas occurring in post-transplantation patients. Am J Surg Pathol.

[54] Herreman A, Dierickx D, Morscio J (2013). Clinicopathological characteristics of posttransplant lymphoproliferative disorders of T-cell origin: Single-center series of nine cases and meta-analysis of 147 reported cases. Leuk Lymphoma.

[55] Gorochov G, Debre P, Leblond V, Sadat-Sowti B, Sigaux F, Autran B (1994). Oligoclonal expansion of CD8+ CD57+ T cells with restricted T-cell receptor beta chain variability after bone marrow transplantation. Blood.

[56] Dharnidharka VR, Douglas VK, Hunger SP, Fennell RS (2004). Hodgkin's lymphoma after post-transplant lymphoproliferative disease in a renal transplant recipient. Pediatr Transplant.

[57] Locker J, Nalesnik M (1989). Molecular genetic analysis of lymphoid tumors arising after organ transplantation. Am J Pathol.

[58] Nelson BP, Locker J, Nalesnik MA, Fung JJ, Swerdlow SH (1998). Clonal and morphological variation in a posttransplant lymphoproliferative disorder: Evolution from clonal T-cell to clonal B-cell predominance. Hum pathol.

[59] Ibrahim HA, Menasce LP, Pomplun S, Burke M, Bower M, Naresh KN (2011). Presence of monoclonal T-cell populations in B-cell post-transplant lymphoproliferative disorders. Mod Pathol.

[60] Dotti G, Fiocchi R, Motta T (2000). Epstein-Barr virus-negative lymphoproliferative disorders in long-term survivors after heart, kidney, and liver transplant. Transplantation.

[61] Martin A, Baran-Marzak F, El Mansouri S (2000). Expression of p16/INK4a in posttransplantation lymphoproliferative disorders. Am J Pathol.

[62] Hoshida Y, Hongyo T, Nakatsuka SI (2002). Gene mutations in lymphoproliferative disorders of T and NK/T cell phenotypes developing in renal transplant patients. Lab Investig.

